# Synaptic potentiation requires PARP1 activation: prevailing concepts are revisited

**DOI:** 10.1038/s41380-025-03426-x

**Published:** 2026-01-06

**Authors:** Malka Cohen-Armon, Menahem Segal

**Affiliations:** 1 Gray Faculty of Medicine and Health Sciences & Sagol School of Neuroscience, Tel-Aviv University, Tel-Aviv, Israel; 2 Brain Sciences, Weizmann Institute of Science, Rehovot, Israel

**Keywords:** Neuroscience, Physiology

## Abstract

The therapeutic potential of PARP inhibitors in neurodegenerative diseases of the central nervous system is widely accepted. This prevailing concept is challenged by more recent findings, unveiling the role of PARP1 activity in synaptic long-term potentiation, synaptic plasticity and long-term memory. So, PARP1 inhibition, though intended to prevent memory deterioration, actually interferes with stimulation-induced synaptic plasticity and long-term memory. On the other hand, PARP inhibitors could be useful for preventing mental disorders associated with memory retrieval.

PARP1, polyADP-ribose polymerase1, is an abundant nuclear protein, highly conserved in eukaryotes, except yeast [1, 2]. PARP1 is a member of a group of ADP-ribose transferases that bind ADP-ribose (Adenosine-diphosphate-ribose) derived from NAD^+^ (nicotine amide- dinucleotide). ADP-riboses are bound covalently to PARP proteins and to their substrates [1–4]. Activated PARP1 cleaves NAD^+^ into nicotine and ADP-ribose. ADP-riboses construct long, negatively charged ADP-ribose polymers (PAR) via glycoside bonds [1–4]. This covalent post translational modification of proteins by polyADP-ribosylation is implicated in DNA repair as well as, in a variety of epigenetic mechanisms [1–10]. Substrates of PARP1 include linker histone H1, core histones and chromatin-structure modifiers [3–10]. The post-translational modification of chromatin-bound proteins by polyADP-ribosylation is critical for genome stability, DNA repair, chromatin remodeling, DNA replication and gene expression [1–10]. In addition, ADP-riboses bind non-covalently to *macro* domains, which are conserved modules with a high‐affinity for ADP‐ribose, including ADP-ribose polymers, either free or bound to proteins [11].

PARP1 is implicated in various forms of DNA repair, including single strand breaks repair, double strand breaks repair and base excision repair [1–5]. In the repair of DNA single-strand breaks, PARP1 bound to DNA via zinc fingers in its DNA-binding domain, is activated and polyADP-ribosylated [1–3]. Binding of the constructed ADP-ribose polymers to *macro* domains in the scaffold protein XCCR1 (X-ray repair cross-complementing protein 1) is implicated in the recruitment of DNA-repair enzymes to the DNA breaks [12, 13]. In addition, PARP1 induced polyADP-ribosylation of chromatin bound proteins adjacent to the DNA break causes local chromatin relaxation, due to repulsion between the negatively charged polyADP-ribosylated proteins in the chromatin and the negatively charged DNA [1–6]. The transient eviction of polyADP-ribosylated linker histone H1 mainly contributes to local chromatin relaxation [14, 15], which is a necessary step in DNA repair and DNA transcription [2, 5–8, 14, 15].

PolyADP-ribosylation of PARP1 is a transient modification. PARG, polyADP-ribose glycohydrolase [16, 17] exerts both endoglycosylase and exoglycosylase activity that hydrolyse the ribose-ribose bonds in the PAR chains of polyADP-ribosylated PARP1 [6, 17]. This activity of PARG restores free ADP-riboses and restores the DNA binding potency of PARP1 free from negatively charged ADP-ribose polymers [1–3, 6, 17]. Single strand breaks in the DNA are constantly repaired under physiological conditions [1–3]. However, highly activated PARP1, under conditions causing a massive damage to the DNA, promotes cell death [18, 19]. In this cell-death mechanism, polyADP-ribose polymers are released from the nucleus to the cytosol and induce the release of apoptosis-inducing factor (AIF) from the mitochondria [18, 19]. AIF promotes a caspase-independent cell death (Parthanatos) [18, 19]. In this mechanism, AIF binds to macrophage migration inhibitory factor (MIF) that acts in the nucleus as DNA nuclease, causing unrepairable DNA fragmentation [18, 19]. In addition, intense polyADP-ribosylation in cells with massively damaged DNA, promotes cell death due to NAD^+^ consumption [18–20]. NAD^+^ is produced in the electron transport chain for ATP generation in the mitochondria [20]. Declining levels of ATP due to high NAD^+^ consumption promote cell death [20].

DNA breaks could result from free radicals reacting with the bases and sugar moieties in the DNA, thereby disrupting the covalent bonds in the DNA strand [21–23]. Free O_2_^-^ radicals are moderately produced in the mitochondria, even under physiological conditions [20]. Their production is intensified by mitochondria dysfunction under oxidative stress, or in various pathological conditions [20–23]. ONOO^-^ radicals are produced by O_2_^-^ radicals reacting with NO (nitric oxide), which is massively secreted in response to inflammation [24, 25]. Cell death of cerebral neurons under pathological conditions has been attributed to an extensively damaged DNA, caused by oxidative stress due to mitochondrial dysfunction or hypoxia injury usually caused by stroke, or inflammation [26, 27]. Microglia and astrocytes produce large amounts of NO during inflammation, causing vasodilation, which promotes healing by leukocyte transmigration into the extravascular space [24, 25]. However, high levels of NO that exacerbate neuro-inflammation [24, 26, 27], cause extensive DNA damage that leads to cell death [25, 27]. Down-regulation of nitric oxide synthases (NOS) has been one of the suggested remedies for ameliorating cell death in neurodegenerative diseases [24, 27]. However, since NO secretion also controls neuronal activity and cognitive functions under physiological conditions [24], treatments with NOS inhibitors became controversial [27].

Progression of cell death by Parthanatos [18, 19], and the implication of PARP1 in the expression of pro-inflammatory genes [26–28], turned PARP1 inhibition into a promising mechanism for decreasing cell death caused by severely damaged DNA in neurodegenerative diseases [27]. This widely accepted therapeutic potential of PARP1 inhibitors in neurodegenera-tion causing cognitive impairment [27, 29], is challenged by more recent findings. These findings unveiled the role of PARP1 polyADP-ribosylation in long-term synaptic potentiation, synaptic plasticity, and in long term memory formation during learning [30–32].

## PARP1 polyADP-ribosylation is required for long-term synaptic potentiation and memory

**Fig. 1:**
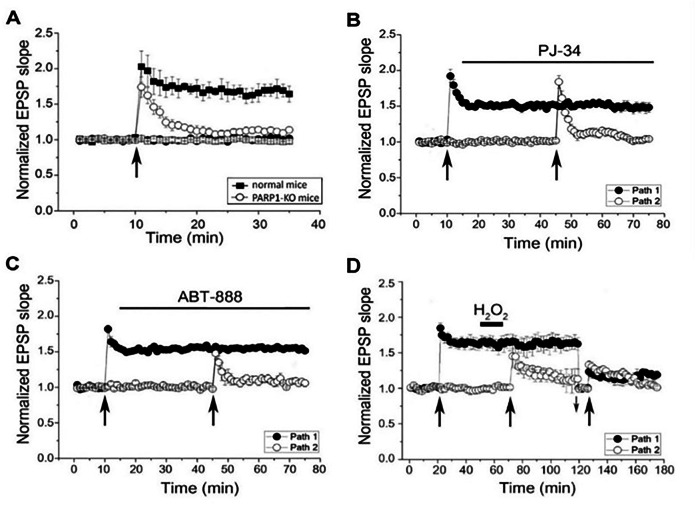
PARP1 polyADP-ribosylation is required for LTP generation. Field excitatory postsynaptic potentials (fEPSPs) were recorded from hippocampal slices. Normal LTP was measured in the hippocampal CA3-CA1 connections of WT mice in response to a tetanic stimulation (100 Hz, 1 sec) (●). **A** LTP was not generated by the same stimulation in PARP1 KO mice (○). (**B, C**) The high-frequency stimulation was delivered to each of 2 independent pathways. PARP inhibitors PJ34 (**B**) and ABT-888 (**C**) prevented LTP generation in these connections of WT mice (○). Tetanic stimulation before application of the PARP1 inhibitors PJ34 and ABT-888 generated a sustained LTP (●). PJ-34 and ABT-888 did not affect the baseline activity, nor the already potentiated responses (LTP), while they completely prevented the generation of novel LTP. Upward arrows indicate applied electrical stimulation. (**D**) DNA single-strand breaks prevent the generation of LTP. The high-frequency stimulation delivered to each of 2 independent stimulation-recording pathways generated LTP (●). The same stimulation delivered to one pathway 15 min after application of H_2_O_2_ (1 mM, 10 min, and 5 min. washout), failed to produce LTP (○). Similarly, the same stimulation delivered to each pathway 80 min after H_2_O_2_ application and adjustment of the stimulation intensity to baseline level (downward small arrow) failed to induce LTP of both pathways (○, ●). (From ref. 30).

Long-term memory of trained animals was prevented by PARP inhibition, while their short-term memory was not impaired [31, 32]. To understand the underlying molecular mechanism that implicates PARP1 activation in memory, we examined the effect of various stimulations on the activation of PARP1 in cortical and hippocampal neurons in primary cell cultures [30]. PARP1 was activated by high frequency electrical stimulation that generated a long-term synaptic potentiation (LTP) [30] (Fig. 1). This stimulation also induced expression of immediate early genes (IEG) *cfos, zif* and *arc*, which are implicated in synaptic plasticity [33, 34]. The expression of these genes was extensively downregulated by PARP inhibition, or after PARP1 silencing [30]. It was also extensively down regulated in cultured neurons prepared from PARP1-KO mice, i.e., after PARP1 genetic deletion [30]. Furthermore, an alternative mode of PARP1 activation in the absence of DNA damage has been identified [8]. In cell-free systems, recombinant PARP1 bound to recombinant phosphorylated Erk2 was poly-ADPribosylated in the presence of NAD^+^ [8]. In rodent hippocampal and cortical neurons, PARP1 was activated by binding to phosphorylated Erk2 that translocated into the nucleus in response to a variety of stimulations activating the MAP-kinase phosphorylation cascade [35–37]. Moreover, consensus docking sites of Erk were identified in the HD and WGR domains of PARP1 [30, 38, 39]. Intramolecular modulations in PARP1 and phosphorylated Erk2 following their binding were analyzed by using the anisotropic network model (ANM, http://ignmtest.ccbb.pitt.edu/cgi-bin/anm/anm1.cgi) [40]. The identified intramolecular structural changes in PARP1 bound to phosphorylated Erk2 included movements of the helical domain (HD) and the catalytic domain (CAT) of PARP1 in opposite directions, exposing the NAD^+^ binding site in the CAT domain of PARP1 [30, 41]. In support, structural analysis has implicated the HD domain of PARP1 in the NAD^+^ binding to its site in the catalytic domain of PARP1 [42]. Exposure of the NAD^+^ binding site in PARP1-bound to phosphorylated Erk2 was in line with the high affinity of NAD^+^ for PARP1-bound to phosphorylated Erk2. About 70-times higher affinity than the affinity of NAD^+^ for DNA-bound PARP1 [8, 30]. This DNA-independent mode of PARP1 activation in response to signal transduction mechanisms activating the MAP-kinase phosphorylation cascade, implicates PARP1 activation in a variety of physiological processes inducing gene expression [37]. Notably, PARP1 polyADP-ribosylation did not involve PARP1 phosphorylation by phosphorylated Erk2 [8]. In cell-free systems, recombinant PARP1 was similarly polyADP-ribosylated by binding to either active or inactive recombinants of phosphorylated Erk2 [8]. Furthermore, the resulting polyADP-ribosylation of PARP1 bound to phosphorylated Erk2 did not interfere with the binding of phosphorylated Erk2 to the Erk docking sites in PARP1 [8]. Thus, polyADP-ribosylation of PARP1 could last as long as PARP1 is bound to phosphorylated Erk2 [8, 30, 43]. Also, the binding of phosphorylated Erk2 to PARP1 did not interfere with the activity of either PARP1 or phosphorylated Erk2 [8, 30]. One of the prominent substrates of PARP1, linker histone H1 was polyADP-ribosylated by PARP1-bound to phosphorylated Erk2, both in cell free systems, and in stimulated cultured cerebral neurons [8, 30]. Poirier and colleges first demonstrated the relaxation of the condensed chromatin structure in response to polyADP-ribosylation of histone H1 [14]. Eviction of polyADP-ribosylated H1 in response to stimulation inducing PARP1 polyADP-ribosylation, has been demonstrated in depolarized cerebral neurons [44]. Erk2 phosphorylation, PARP1 and H1 polyADP-ribosylation and phosphorylation of transcription factor Elk1, a prominent substrate of Erks [45], were measured in cultured cerebral neurons stimulated by a high frequency electrical stimulation that induces synaptic long-term potentiation (LTP) [30, 45–47]. In addition, acetylation of histone H4 and the expression of IEG *cfos, zif* and *arc* that are implicated in synaptic plasticity, were measured in the stimulated cultured cerebral neurons [30, 33, 34]. In support, the insertion of recombinant phosphorylated Erk2 into transiently permeabilized cortical neurons, similarly caused PARP1 polyADP-ribosylation, Elk1 phosphorylation and histone acetylation [8]. Elk1 phosphorylation, inducing the HAT (histone acetyltransferase) activation of CBP (CREB binding protein) and histone acetylation mediated IEG expression [30, 37, 47–50]. These identified molecular mechanisms outline a stimulation-induced Erk2 phosphorylation, causing polyADP-ribosylation of PARP1 that “paves the way” in the chromatin for phosphorylation of transcription factors by phosphorylated Erk2- bound to polyADP-ribosylated PARP1. In this mechanism, local chromatin relaxation by a brief release of polyADP-ribosylated histone H1 from the DNA, mediates IEG expression [8, 30, 44, 49, 50].

Furthermore, the possible implication of PARP1-polyADP-ribosylation in synaptic plasticity was assessed by measuring its effect on long-term synaptic potentiation (LTP) [46, 51]. LTP was generated in response to high frequency stimulation of the hippocampal CA3-CA1 connections, and it lasted for hours [30] (Fig. 1). PARP1, polyADP-ribosylation and phosphorylated Erk2 were necessary for LTP generation [30, 52]. MEK inhibitors, preventing Erk2 phosphorylation, prevented the generation of LTP [30]. LTP was not generated in the hippocampal CA3-CA1 connections of PARP1 genetically deficient mice (i.e. in the hippocampus of PARP1 KO mice) [30] (Fig. 1A). PARP1 inhibitors applied before stimulation prevented the generation of LTP in the hippocampal CA3-CA1 connections of wild-type (WT) mice [30] (Fig. 1B and C), whereas PARP1 inhibitors applied after LTP generation, did not affect the already generated LTP [30], (Fig. 1B and C). In addition, the applied PARP inhibitors did not affect the stimulation-induced post synaptic excitatory currents [30]. These results implicated PARP1 polyADP-ribosylation in the generation of synaptic long-term potentiation and synaptic plasticity [30, 46, 51].

Furthermore, PARP polyADP-ribosylation was required for learning abilities of the marine slug *Aplysia*, and for the learning abilities of rodents [31, 32]. PolyADP-ribosylation of both PARP1 and histone H1 was measured only in the buccal ganglia of *Aplysia* after training to avoid inedible food [31]. In addition, *Aplysia* did not acquire long-term memory during training in the presence of PARP1 inhibitors applied before training [31]. Their long-term memory was not impaired by PARP inhibitors applied after training [31]. Also, their acquired short memory was not impaired by PARP1 inhibition [31]. PARP inhibitors also prevented long-term memory formation in mammals. Mice were trained for object recognition or trained to avoid frightening experiences by fear conditioning [32]. Both PARP1 and histone H1 were polyADP-ribosylated in their brain cortex and hippocampus after the training [32]. In addition, their long-term memory was impaired when they were injected with PARP inhibitors 30 min before the training [32]. However, PARP inhibitors injected immediately after their training did not impair their long-term memory [32]. Their acquired short-term memory was not impaired by PARP inhibitors, injected either before or after training [32]. These results indicated a dependence of long-term memory formation during training on polyADP-ribosylation [31, 32], and they further support the dependence of LTP generation and IEG expression on the Erk2-induced PARP1 polyADP-ribosylation [30] (Fig. 1A-C). In this mechanism of PARP1 activation, PARP1 polyADP-ribosylation was independent of DNA breaks [8, 30]. Moreover, DNA damage interfered with the polyADP-ribosylation dependent LTP generation (Fig. 1D), as well as with IEG expression in the stimulated cerebral neurons [30].

Learning has been associated with the expression of immediate early genes [33, 34, 49–51, 53, 54]. This is in accordance with evidence associating a fast IEG expression during learning with the identified Pol II stalled just downstream of the IEG transcription start site in rat cerebral neuron [55]. Epigenetic mechanisms associated with learning include the implication of the HAT activity of CBP, and histone acetylation occurring during learning [56–58]. In general, the activity of HATs was necessary for memory acquisition, while the activity of histone deacetylases (HDHC) interfered with memory consolidation [57, 58]. Methylation of core histones and DNA methylation have been associated with cognitive abilities [59, 60]. Chromatin remodeling has been also implicated in learning. Chromatin remodeling by the insulator protein CTCF affecting gene expression and silencing, has been implicated in long-term memory [9, 60, 61]. Mutations in CTCF have been found in individuals with intellectual disability [62]. CTCF is one of the targets of PARP1 [9]. PolyADP-ribosylation of CTCF transiently exposes regions in the DNA to transcription by altering the binding sites of CTCF in the DNA [9]. In another mechanism altering DNA transcribing sites, the helicase activity of the chromatin remodeling complex BAF (Brg l /h Brm associated factor) has been implicated in cognitive abilities [63].

## LTP induction is prevented in the presence of damaged DNA

**Fig. 2:**
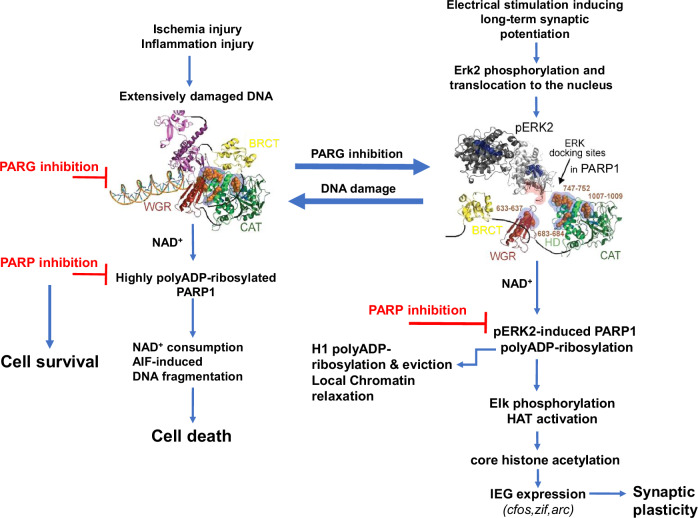
Two flow charts are presented. **Left**- Cell death or survival, depending on the intensity of polyADP-ribosylation of PARP1 bound to damaged DNA. **Right**- signal-transduction mechanism implicating phosphorylated Erk2-induced PARP1 polyADP-ribosylation in stimulation-induced expression of immediate early genes (IEG). **Left**: Cell death in cells with massively damaged DNA, caused by highly polyADP-ribosylated PARP1 is prevented by PARP inhibition or by PARG inhibition that prevents the recurrent binding of PARP1 to the damaged DNA. **Right**: Phosphorylated Erk2 binding to Erk consensus sites in PARP1 in response to stimulation generating LTP, leads to PARP1 polyADP-ribosylation and histone H1 polyADP-ribosylation and eviction, mediating phosphorylation of transcription factor Elk1 by PARP1-bound phosphorylated-Erk2 (pERK2). Elk1 phosphorylation of CBP induces its HAT activation, core histone acetylation and IEG (*cfos, zif, arc*) expression. Occlusion of Erk docking sites in PARP1 bound to DNA breaks (**Left chart**) or PARP1 inhibition prevent IEG expression. Treatment with PARG inhibitors which prevents a recurrent binding of PARP1 to DNA, preserves IEG expression and synaptic plasticity in the presence of damaged DNA. (the PARP1 structures are included in ref. 30).

DNA damage interference with IEG expression [8, 30] is in line with the interference of DNA damage with the generation of LTP in the hippocampus of mice [30], (Fig. 1D). PolyADP-ribosylation of histone H1, Elk1 phosphorylation and the expression of IEG *cfos, zif* and *arc* were prevented in the presence of damaged DNA [30]. This effect of damaged DNA was attributable to the interference of DNA damage with PARP1-binding to phosphorylated Erk2 in the stimulated neurons [30]. In support, in cell free systems, recombinant phosphorylated Erk2 did not bind to recombinant PARP1 in the presence of damaged DNA [8]. This effect of DNA damage on PARP1 binding to phosphorylated Erk2 is attributed to structural modifications in PARP1 bound to DNA breaks [30]. The BRCT domain of PARP1, which is implicated in the binding of DNA-bound PARP1 to XRRC1 during DNA repair, occludes the consensus docking sites of Erk in the HD and WGR domains of PARP1 bound to DNA [12, 30, 38, 39, 64] (Fig. 2). In accordance, stimulation-induced IEG expression was measured in the presence of damaged DNA in cortical neurons expressing truncated PARP1, missing its DNA-binding domain [30, 64]. Thus, binding of phosphorylated Erk2 to the consensus Erk docking sites in the HD and WGR domains of PARP1 is a necessary step enabling PARP1 polyADP-ribosylation and chromatin relaxation, preceding IEG expression in response to stimulation activating the MAP-kinase phosphorylation cascade [30, 37]. In accordance, PARP1 binding to damaged DNA, interfering with its binding to phosphorylated Erk2, prevented synaptic long-term potentiation and the expression of IEG that are implicated in synaptic plasticity [30]. To avoid the interference of damaged DNA with IEG expression, stimulated cell cultures of cortical and hippocampal neurons with damaged DNA were treated with the PARG inhibitor, Gallotannin that prevents the recurrent binding of PARP1 to the damaged DNA [1–5, 17, 30, 65]. IEG expression was measured in these treated neuronal cell cultures even in the presence of DNA damage [30], suggesting that stimulation-induced synaptic plasticity could be preserved even in the presence of damaged DNA (Fig. 2).

Evidence for interference of DNA damage with synaptic plasticity is in line with the experienced cognitive disabilities described as “Brain-Fog” or “Chemo-Brain”, during and after treatments with DNA damaging cancer therapy, including cancer therapy based on PARP inhibitors [66–69]. The working memory, visual memory, and verbal memory are usually impaired in patients experiencing “brain-fog” [66, 67]. A similar deterioration in cognitive abilities was observed in tumor-free mice treated with DNA damaging agents, suggesting that the cognitive disabilities are not related to the cancer disease [66].

DNA damage in neuronal and glial cells, and memory deterioration are both hallmarks of neurodegeneration in the central nervous system [27]. DNA damage and cognitive disabilities have been attributed to the accumulation of neurotoxic protein aggregates [27, 70–75]. The pathogenesis of Alzheimer’s disease involves the accumulation of two neurotoxic protein aggregates in the central nervous system: amyloid-β (Aβ) peptide and hyper-phosphorylated tau proteins, both causing DNA damage, neuro-inflammation and deterioration of cognitive abilities [71–75]. Thus, PARP1 inhibition seems a reasonable remedy for ameliorating the rate of cell death caused by high levels of PARP1 polyADP-ribosylation in cells with damaged DNA [18, 19, 27, 72–74]. However, in view of the role of PARP1 activity in synaptic potentiation, synaptic plasticity and long-term memory [30–32], (Fig. 1), this widely accepted potential of PARP1 inhibitors is revisited. PARP inhibitors in the same concentration range, efficiently interfere with synaptic plasticity and long-term memory acquisition during learning [18, 30, 31]. Thus, although the treatment with PARP inhibitors is beneficial in attenuating cell death in neurodegenerative diseases [27, 72–74], it can also prevent the long-term potentiation of still functioning synapses in undamaged brain regions, thereby promoting neuro-degeneration by preventing neuronal activity [76–79].

To avoid the hyper-activation of PARP1 in the presence of damaged DNA without impairing synaptic plasticity, PARP1 inhibitors can be replaced by PARG inhibitors [30] (Fig. 2). In cultured cerebral neurons, PARG inhibition by Gallotannins spared IEG expression in the presence of DNA breaks by preventing the binding of PARP1 to the damaged DNA [30]. Since polyADP-ribosylation does not interfere with the binding of phosphorylated Erk2 to the Erk consensus sites in PARP1 [8, 30], the treatment with Gallotannin can spare the stimulation-induced binding of polyADP-ribosylated PARP1 to phosphorylated Erk2 in the presence of damaged DNA [8, 30]. The following chromatin relaxation and Elk1 phosphorylation promoting the expression of IEG *cfos, zif* and *arc* were spared in stimulated cultured cortical and hippocampal neurons under hypoxia or in the presence of DNA damaging agent [30]. This molecular mechanism might spare synaptic plasticity in neurodegenerative diseases, as well. The replacement of PARP inhibitors by PARG inhibitors might spare cognitive abilities, while preventing cell death by highly polyADP-ribosylated PARP1 in neurodegenerative diseases (Fig. 2). In support, Tannins were reported to be beneficial in neuroprotection as well as, in ameliorating neurodegeneration [80–86]. Tannic acid prevented cognitive impairment in the β-amyloid animal model (APP/PS1) [82], and Inhibition of β-secretase 1 was an additional beneficial effect of tannic acid [82].

While the therapeutic effect of PARP inhibitors in neurodegeneration is revisited, the identified role of PARP1 activation in long-term memory [30–32] (Fig. 1) could be useful for treating psychological disorders associated with the retrieval of devastating memories.

PARP inhibitors could be useful in preventing the retrieval of traumatic memory [87, 88], and in preventing the retrieval of reward-related memory causing craving for drugs in addicts or leading to uncontrolled compulsive behavior [89–91]. In view of these encouraging evidence, possible interference of PARP1 inhibitors with memory retrieval deserves further attention.

In summary, the identified role of PARP1 activation in LTP generation and in long-term memory opens new potential avenues for therapy in both neurodegeneration and mental disorders.
